# Trifluridine/Tipiracil Plus Bevacizumab Versus Regorafenib in Elderly Patients with Refractory Metastatic Colorectal Cancer: A Real-World Comparative Study

**DOI:** 10.3390/cancers18050788

**Published:** 2026-02-28

**Authors:** Yu-Kang Tseng, Chang-Lin Lin, Shih-Wei Chiang, Feng-Fan Chiang

**Affiliations:** Division of Colorectal Surgery, Department of Surgery, Taichung Veterans General Hospital, Taichung 40705, Taiwan; 10510ken@gmail.com (Y.-K.T.);

**Keywords:** metastatic colorectal cancer, elderly, trifluridine/tipiracil, regorafenib, real-world study

## Abstract

Background: Taiwan’s transition to a super-aged society presents challenges in managing elderly patients with refractory metastatic colorectal cancer (mCRC). This study evaluated the real-world effectiveness of trifluridine/tipiracil (FTD/TPI) plus bevacizumab versus regorafenib in this specific geriatric population. Methods: A retrospective study analyzed patients aged ≥ 65 treated between 2019 and 2023. Patients received regorafenib (*n* = 46) or FTD/TPI plus bevacizumab (*n* = 35). Primary endpoints included overall survival (OS) and time to treatment failure (TTF). Results: FTD/TPI plus bevacizumab was associated with significantly longer median OS compared to regorafenib (12.5 vs. 6.5 months; *p* = 0.039). In multivariate analysis, the combination regimen was identified as an independent predictor of favorable TTF (adjusted HR 0.44, 95% CI 0.22–0.91, *p* = 0.028). Regorafenib was associated with symptomatic toxicities like hand–foot skin reaction (58.7%), while the combination primarily caused asymptomatic neutropenia. Crucially, permanent discontinuation due to adverse events was significantly lower in the combination arm (2.9% vs. 30.4%, *p* < 0.001), facilitating a higher proportion of patients receiving subsequent systemic therapy (45.7% vs. 17.4%, *p* = 0.011). Conclusions: FTD/TPI plus bevacizumab is associated with improved survival outcomes and better tolerability than regorafenib. By demonstrating a manageable safety profile and minimizing treatment attrition, this regimen ensures a sustainable continuum of care, representing a favorable therapeutic option for elderly patients.

## Simple Summary

As society ages, treating elderly patients with advanced colorectal cancer becomes increasingly challenging due to their fragility and intolerance to toxic side effects. This study compared two standard late-line treatments—trifluridine/tipiracil (FTD/TPI) combined with bevacizumab versus regorafenib—specifically in elderly Asian patients. We analyzed real-world data to determine which therapy offers better survival and safety. Our findings suggest that the combination of FTD/TPI plus bevacizumab was associated with significantly longer overall survival (12.5 vs. 6.5 months) compared to regorafenib. Crucially, the combination therapy had milder side effects and significantly lower treatment discontinuation rates, allowing elderly patients to continue therapy and receive subsequent treatments. This study highlights the importance of selecting tolerable treatments to maximize survival outcomes in this vulnerable population.

## 1. Introduction

The global burden of colorectal cancer (CRC) continues to rise, with a significant proportion of cases diagnosed in the elderly population [1]. This demographic shift is particularly critical in Taiwan, which transitioned into a “super-aged society” in 2025, with individuals aged 65 and older accounting for more than 20% of the total population. As the number of elderly patients with metastatic colorectal cancer (mCRC) increases, clinicians are increasingly faced with the challenge of balancing treatment efficacy against the heightened physiological vulnerability and reduced functional reserve of this age group [2,3].

For patients with refractory mCRC who have progressed after standard therapies involving fluoropyrimidines, oxaliplatin, and irinotecan, the emergence of regorafenib and trifluridine/tipiracil (FTD/TPI) has provided essential salvage-line options. For over a decade, regorafenib has remained a cornerstone of late-line therapy. Based on the findings of the landmark CORRECT and CONCUR trials, regorafenib is primarily utilized as a monotherapy in clinical practice [4,5]. However, its real-world utility in the geriatric cohort is often hampered by a high incidence of symptomatic toxicities, which frequently lead to treatment interruptions or premature discontinuation [6].

More recently, the combination of FTD/TPI plus bevacizumab has established a new standard of care. The pivotal SUNLIGHT trial demonstrated that adding bevacizumab to FTD/TPI significantly prolonged overall survival (OS) and progression-free survival (PFS) compared to FTD/TPI monotherapy, while maintaining a manageable safety profile [7]. Consequently, this combination regimen has been widely adopted as a preferred third-line or later-line strategy [8].

Despite these therapeutic advancements, there is a lack of direct, head-to-head comparisons between regorafenib monotherapy and FTD/TPI plus bevacizumab specifically within the real-world elderly population. Older patients are often underrepresented in phase III clinical trials due to strict inclusion criteria regarding age and comorbidities. Furthermore, data regarding treatment continuity and the impact of specific toxicity profiles on Asian elderly patients remains sparse.

While the SUNLIGHT trial established FTD/TPI plus bevacizumab as a standard therapy, comparative evidence against regorafenib in the geriatric population remains scarce. In Taiwan’s real-world practice, both regimens are actively utilized in the third-line setting. However, the optimal selection for elderly patients—requiring a delicate balance between survival benefits and toxicity—remains unclear due to a lack of specific real-world data. Therefore, this study aims to provide critical comparative evidence to guide clinical decision-making in this vulnerable demographic.

To address this clinical gap, we conducted a retrospective, single-center study at Taichung Veterans General Hospital. This study aims to evaluate and compare the real-world effectiveness and safety of regorafenib monotherapy versus FTD/TPI plus bevacizumab in elderly Asian patients (age ≥ 65). This analysis focuses on the incidence of adverse events, the rate of treatment discontinuation due to toxicities, time to treatment failure (TTF), and overall survival, aiming to provide practical evidence for optimizing the management of refractory mCRC in an increasingly aging society.

## 2. Materials and Methods

This study was designed as a retrospective, single-center observational analysis conducted at Taichung Veterans General Hospital, a tertiary medical center in Taiwan. The research protocol received formal approval from the Institutional Review Board of Taichung Veterans General Hospital (Certificate No. CE251121C) and was performed in strict accordance with the Declaration of Helsinki [9]. Due to the retrospective nature of the data collection and the anonymization of patient identifiers, the requirement for written informed consent was waived by the ethics committee.

The selection of the specific salvage-line regimen was determined through a process of Shared Decision-Making (SDM) between the attending oncologists, the patients, and their caregivers [10]. During clinical consultations, patients were comprehensively informed regarding the expected therapeutic efficacy, potential toxicity profiles, and the administrative schedules of both regorafenib and FTD/TPI plus bevacizumab. The final treatment choice incorporated the physician’s clinical assessment of the patient’s physiological reserve alongside the patient’s personal preferences and quality-of-life priorities. This approach ensured that all elderly participants were fully cognizant of the treatment goals and the risks of treatment-related adverse events prior to commencement.

Regarding dose management, the initial dose of regorafenib was customized at the discretion of the attending oncologists, often starting at 80 mg or 120 mg per day. This approach aligns with the ‘dose-escalation’ strategy validated by the ReDOS trial, which is particularly relevant for elderly patients to minimize initial toxicities while maintaining therapeutic exposure [11]. Dose adjustments for FTD/TPI were primarily based on body surface area (BSA) and renal function, as per standard protocol.

Patients diagnosed with refractory metastatic colorectal cancer (mCRC) who initiated systemic therapy with either regorafenib monotherapy or the combination of trifluridine/tipiracil (FTD/TPI) and bevacizumab between January 2019 and December 2023 were identified for screening.

Inclusion Criteria: To be eligible for the final analysis, patients were required to meet the following criteria: (1) aged 65 years or older at the time of treatment initiation; and (2) receiving the study drugs as a third-line or later-line systemic therapy.

Exclusion Criteria: Potential participants were excluded based on the following pre-specified conditions: (1) presence of multiple primary malignancies; (2) severe deficiency in essential clinical or follow-up data; (3) use of FTD/TPI without concurrent bevacizumab; (4) administration of regorafenib in combination with other cytotoxic chemotherapy; (5) treatment involving the rechallenge of either regorafenib or FTD/TPI; (6) early treatment discontinuation within four weeks for reasons other than disease progression; (7) early mortality occurring within two weeks of treatment commencement; and (8) lack of radiographic or nuclear medicine assessments (e.g., CT, MRI, or PET/CT) for a period exceeding six months during the treatment course, which would preclude accurate evaluation of disease status.

In this study, the combination of FTD/TPI and bevacizumab was defined as the concurrent administration of both agents within a single treatment cycle, consistent with the dosing schedule validated in the SUNLIGHT trial [7]. Specifically, patients were included in the combination group if bevacizumab was initiated either simultaneously with FTD/TPI or within 14 days of the first dose of FTD/TPI. This timeframe was chosen to ensure that the two agents were intended as a synchronized doublet regimen according to clinical practice at our institution.

Comprehensive clinical data were extracted from the institutional electronic medical record (EMR) system. Baseline characteristics included age, sex, body mass index (BMI), ECOG performance status (PS), primary tumor location, and RAS/BRAF mutation status.

Tumor response was evaluated primarily using computed tomography (CT) scans performed approximately every 8 to 12 weeks. Radiographic progression was ascertained by board-certified radiologists and treating physicians in strict accordance with the Response Evaluation Criteria in Solid Tumors (RECIST) version 1.1 guidelines [12]. While biochemical markers (e.g., CEA) were monitored as clinical adjuncts, objective radiographic progression served as the definitive criterion for efficacy assessment. In cases where the determination of progression was clinically ambiguous or complex, the patient’s status was reviewed during weekly multidisciplinary team (MDT) meetings, where a consensus was reached by a panel of colorectal surgeons, medical oncologists, radiologists, and pathologists [13].

Safety was evaluated based on the Common Terminology Criteria for Adverse Events (CTCAE), version 5.0. Symptomatic toxicities, particularly hand–foot skin reaction (HFSR) and gastrointestinal events, as well as laboratory abnormalities such as neutropenia, were documented [14]. Dose modifications, including reductions and treatment interruptions, were implemented based on institutional protocols for toxicity management at the discretion of the treating physicians [4,15].

The primary endpoints were time to treatment failure (TTF) and overall survival (OS). TTF was defined as the interval from the first dose to permanent treatment discontinuation for any reason, including radiological disease progression, symptomatic deterioration, unacceptable toxicity, patient refusal, physician decision, or death. OS was calculated from the date of the first dose until the date of death from any cause or the last follow-up (31 July 2024), at which point patients were censored.

Continuous variables were presented as mean ± standard deviation or median with range, while categorical variables were summarized as frequencies and percentages. Differences between the two treatment groups were compared using Student’s *t*-test or the Mann–Whitney U test for continuous data and the Chi-square test or Fisher’s exact test for categorical data.

Survival outcomes were analyzed using the Kaplan–Meier method and compared via the log-rank test [16]. To identify independent prognostic factors and adjust for potential baseline imbalances, a multivariate Cox proportional hazards model was employed [17]. Covariates such as age, ECOG PS, and metastatic burden were incorporated into the model. All statistical computations were performed using Python (version 3.10) utilizing the lifelines and statsmodels libraries. A two-sided *p*-value of <0.05 was considered statistically significant.

## 3. Results

### 3.1. Patient Characteristics and Disposition

**Figure 1:**
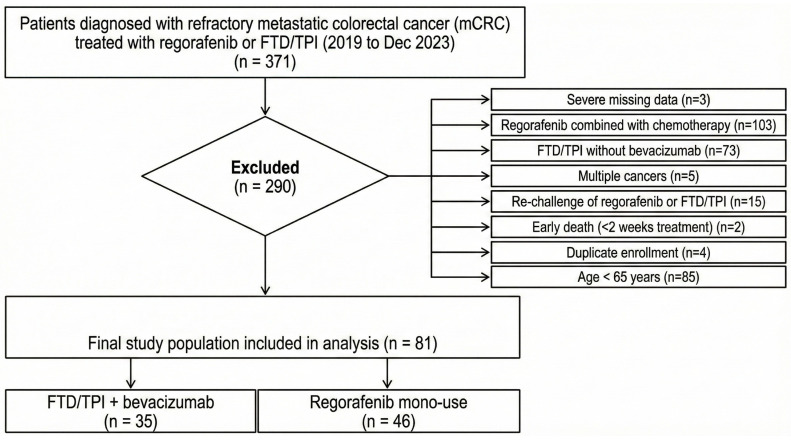
Flowchart of patient selection. A total of 371 patients diagnosed with refractory metastatic colorectal cancer (mCRC) who received either regorafenib or FTD/TPI between January 2019 and December 2023 were initially screened. After applying predefined exclusion criteria, 290 patients were excluded (major reasons included regorafenib combined with chemotherapy [*n* = 103], age < 65 years [*n* = 85], and FTD/TPI without bevacizumab [*n* = 73]). The final study population consisted of 81 elderly patients (aged ≥ 65 years), stratified into the regorafenib monotherapy group (*n* = 46) and the FTD/TPI plus bevacizumab group (*n* = 35). Abbreviations: FTD/TPI, trifluridine/tipiracil; mCRC, metastatic colorectal cancer.

This retrospective, single-center study analyzed data from Taichung Veterans General Hospital. Between 2019 and December 2023, a total of 371 patients diagnosed with refractory metastatic colorectal cancer (mCRC) who received either Regorafenib or Trifluridine/Tipiracil (FTD/TPI) were initially screened for eligibility. Inclusion was limited to patients aged 18 years or older undergoing third-line or later systemic therapy. A sequential exclusion process was applied to 290 patients based on the following criteria: severe data deficiency (*n* = 3), combination of Regorafenib with cytotoxic chemotherapy (*n* = 103), use of FTD/TPI monotherapy without bevacizumab (*n* = 73), presence of multiple primary malignancies (*n* = 5), rechallenge with Regorafenib or FTD/TPI (*n* = 15), early mortality within two weeks of treatment initiation (*n* = 2), duplicate enrollment (*n* = 4), and age younger than 65 years (*n* = 85). The final study cohort comprised 81 elderly patients (age ≥ 65), stratified into the Regorafenib monotherapy group (*n* = 46) and the FTD/TPI plus bevacizumab group (*n* = 35). The selection process is detailed in Figure 1.

Regarding data quality control, specifically in the regorafenib arm, only 2 patients were excluded due to early non-cancer-related death, and another 2 were excluded due to irregular imaging intervals (>6 months) that rendered efficacy inevaluable. These specific exclusions represented <5% of the screened population, minimizing the risk of selection bias.

The final study cohort comprised 81 elderly patients (age ≥ 65), stratified into the Regorafenib monotherapy group (*n* = 46) and the FTD/TPI plus bevacizumab group (*n* = 35).

Demographic and clinical features of the two groups are summarized in Table 1. The study population was well-balanced regarding age, sex, BMI, and ECOG performance status, with most patients presenting an ECOG score of 0–1. No significant differences were observed in comorbidities, primary tumor location, or metastatic burden. However, a significant disparity was noted in prior treatment history; the FTD/TPI plus bevacizumab group had a higher proportion of patients previously treated with anti-EGFR antibodies (48.6% vs. 21.7%, *p* = 0.017) and bevacizumab (97.1% vs. 69.6%, *p* = 0.001).

Regarding post-progression management, a significantly larger proportion of patients in the FTD/TPI plus bevacizumab group proceeded to receive any form of subsequent therapy compared to the regorafenib group (45.7% vs. 17.4%, *p* = 0.007). In the FTD/TPI plus bevacizumab group (*n* = 35), 16 patients (45.7%) received further interventions, primarily driven by systemic crossover to regorafenib (*n* = 14) and ramucirumab-based regimens (*n* = 1). One patient in this group also received local treatment with palliative brain radiation therapy.

In contrast, only 8 patients (17.4%) in the regorafenib group (*n* = 46) underwent subsequent therapy (*p* = 0.011 for systemic treatment alone). Among these, seven patients crossed over to FTD/TPI monotherapy, and one patient received local intervention with radiofrequency ablation (RFA). The markedly lower rate of subsequent treatment in the regorafenib arm suggests that symptomatic toxicities and rapid functional decline often precluded elderly patients from accessing further life-prolonging options. These results reinforce the clinical advantage of the FTD/TPI plus bevacizumab regimen in preserving a “continuum of care” for the geriatric population.

### 3.2. Time to Treatment Failure (TTF) and Survival Outcomes

**Figure 2:**
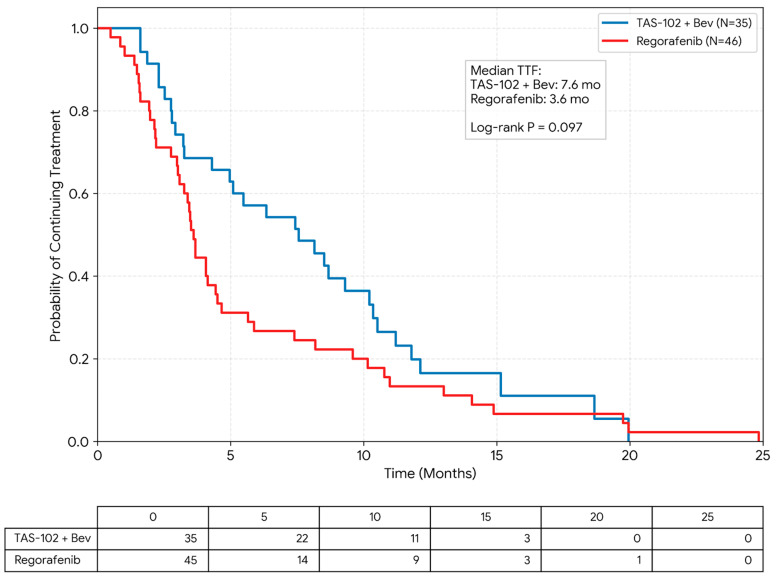
Kaplan–Meier estimates of Time to Treatment Failure (TTF). The median TTF was 7.56 months (95% CI: 4.30–9.30) in the FTD/TPI plus bevacizumab group (blue line, *n* = 35) and 3.61 months (95% CI: 3.02–4.50) in the regorafenib group (red line, *n* = 46). Although the combination therapy demonstrated a clinically meaningful numerical prolongation of treatment duration, the difference did not reach statistical significance according to the univariate log-rank test (*p* = 0.097). Vertical ticks indicate censored data, representing patients who remained on their assigned treatment at the data cutoff date (31 July 2024).

The median TTF was markedly longer for patients receiving FTD/TPI plus bevacizumab than for those receiving regorafenib (7.56 vs. 3.61 months; Figure 2). In the univariate analysis, the combination therapy showed a strong numerical trend toward improved treatment duration, although the difference did not reach the threshold for absolute statistical significance (log-rank *p* = 0.097).

**Figure 3:**
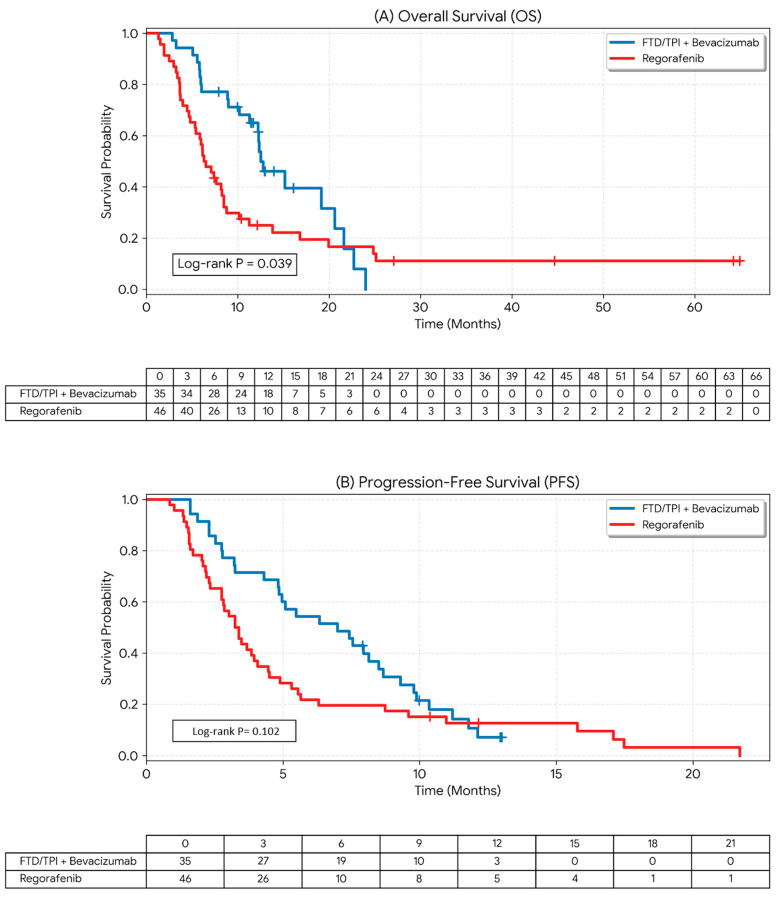
Kaplan–Meier Estimates of Overall Survival and Progression-Free Survival. (**A**) Overall survival (OS) was significantly longer in the FTD/TPI plus bevacizumab group (blue line, *n* = 35) compared to the regorafenib group (red line, *n* = 46), with a median OS of 12.5 months (95% CI: 10.2–20.6) versus 6.5 months (95% CI: 4.8–8.5) (log-rank *p* = 0.039). (**B**) Progression-free survival (PFS) demonstrated a numerical advantage in the FTD/TPI plus bevacizumab group, with a median PFS of 7.0 months (95% CI: 4.8–8.5) versus 3.4 months (95% CI: 2.3–4.1) in the regorafenib group (log-rank *p* = 0.102). Vertical ticks along the curves indicate censored data, representing patients who were alive (for OS) or progression-free (for PFS) at the time of the final data cutoff (31 July 2024). The plateau observed at the end of the OS curve for the combination group reflects long-term survivors within this geriatric cohort.

The FTD/TPI plus bevacizumab group demonstrated a significantly superior median OS compared to the regorafenib group (12.5 vs. 6.5 months; log-rank *p* = 0.039, Figure 3A). The survival curves showed early and sustained separation, with several long-term survivors identified in the combination arm at the time of data cutoff. Regarding disease control, the median PFS was numerically longer in the FTD/TPI plus bevacizumab group compared to the regorafenib group (7.0 vs. 3.4 months), although this trend did not reach statistical significance (log-rank *p* = 0.102, Figure 3B).

### 3.3. Safety Profile and Treatment Continuity

The safety profiles of the two treatment regimens exhibited distinct patterns of toxicity, as summarized in Table 2. Although the overall incidence of any-grade adverse events was comparable between the Regorafenib and FTD/TPI plus bevacizumab groups (82.6% vs. 88.6%, *p* = 0.539), the nature and severity of these events differed significantly.

The FTD/TPI plus bevacizumab group experienced a markedly higher burden of hematologic toxicities. Neutropenia was the most prevalent event in this arm, occurring in 48.6% of patients, with a substantial 31.4% reaching Grade 3 or higher (*p* < 0.001). Furthermore, neutropenic fever was exclusively observed in the combination arm (14.3%, all Grade 3; *p* = 0.013). Other hematologic events, including anemia (5.7%), thrombocytopenia (5.7%), and leukopenia (5.7%), were also noted in the combination group, though these were generally low-grade and clinically manageable. In contrast, the Regorafenib group demonstrated minimal hematologic impact, with no reported cases of neutropenia or leukopenia.

Specifically, febrile neutropenia occurred in 5 patients (14.3%) in the FTD/TPI plus bevacizumab group. All 5 cases required hospital admission and received therapeutic granulocyte colony-stimulating factor (G-CSF) support. No treatment-related deaths occurred. Following recovery, dose reduction was implemented in one patient (from a total daily dose of 120 mg to 50 mg) to prevent recurrence, while the others resumed therapy under close surveillance.

Conversely, the Regorafenib arm was characterized by a high frequency of symptomatic non-hematologic toxicities. Hand–foot skin reaction (HFSR) was the most prominent adverse event, affecting 58.7% of the patients, with 19.6% experiencing Grade 3 severity (*p* < 0.001). Regarding the regorafenib arm, laboratory safety monitoring (including complete blood counts and liver function tests) was conducted every 2 to 4 weeks. This frequency aligns with standard labeling recommendations (bi-weekly for the first two months, then monthly), ensuring that the observed low rate of hematologic toxicity reflects a genuine safety profile rather than under-monitoring.

Non-hematologic toxicities also differed significantly between the two groups. Diarrhea and liver dysfunction were observed exclusively or predominantly in the Regorafenib arm. Diarrhea occurred in 19.6% of Regorafenib-treated patients compared to 0.0% in the combination group (*p* = 0.009). Similarly, liver dysfunction was significantly more frequent with Regorafenib (17.4%; Grade ≥ 3: 8.7%) than with FTD/TPI plus bevacizumab, where no such events were recorded (*p* = 0.009). Regarding other common adverse events, fatigue was prevalent in both arms (43.5% vs. 40.0%). Hypertension (4.3%) was noted in the Regorafenib group, while nausea/vomiting (20.0%) was a primary concern in the FTD/TPI plus bevacizumab group, though Grade ≥ 3 events for these toxicities were rare.

Regarding treatment exposure and dosing patterns, distinct strategies were observed. In the regorafenib arm, the median duration of therapy was 3.7 months (range 0.5–24.8). Reflecting a proactive toxicity management strategy, the majority of patients (69.6%, *n* = 32) initiated treatment at a reduced dose of 80 mg/day, with a stepwise escalation plan. However, given the geriatric profile of our cohort, the maximum tolerated dose was frequently capped at 120 mg/day. Consequently, most patients were maintained at this dose level rather than escalating to the standard 160 mg/day, balancing efficacy with tolerability. Despite this cautious approach, 45.7% of patients still required further dose modifications or interruptions due to adverse events.

In the combination arm, the median duration of therapy was significantly longer at 6.3 months (range 1.6–19.9). Bevacizumab was administered at a standard biological dose of 5 mg/kg every 2 weeks. Dosing adherence for oral FTD/TPI was high, supported by the low rate of dose reductions (11.4%) and minimal discontinuation due to toxicity (2.9%). The median starting dose intensity for FTD/TPI was approximately 100 mg/day (range 50–160 mg/day), consistent with standard body surface area-based dosing.

In summary, the toxicity profile of Regorafenib was dominated by symptomatic, patient-reported cutaneous and gastrointestinal events, along with a significant incidence of hepatotoxicity, whereas the FTD/TPI plus bevacizumab regimen was primarily associated with asymptomatic laboratory abnormalities, specifically neutropenia.

**Figure 4:**
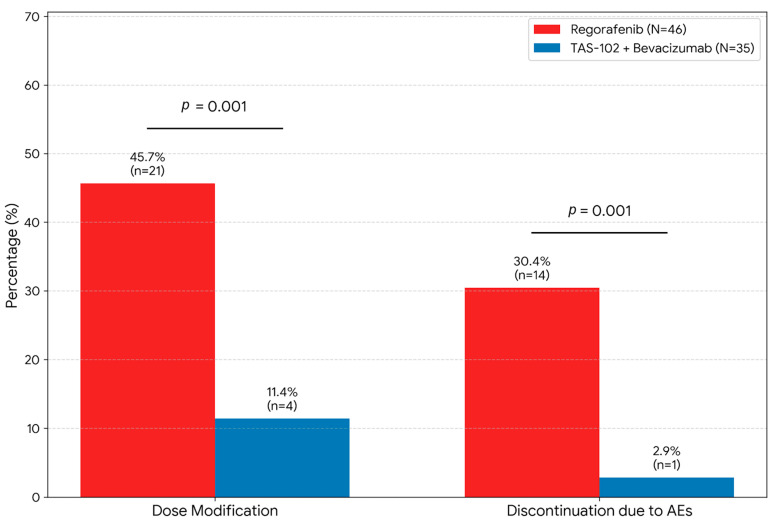
Impact of adverse events on treatment modification and discontinuation. The bar chart illustrates the percentage of patients requiring dose modification (including dose reduction or temporary interruption) and those experiencing permanent discontinuation due to treatment-related adverse events (AEs). The rate of dose modification was significantly higher in the Regorafenib group (red bar, *n* = 46) compared to the TAS-102/Bevacizumab group (blue bar, *n* = 35) (45.7% vs. 11.4%, respectively; *p* < 0.001). Similarly, the incidence of discontinuation due to AEs was strikingly higher for Regorafenib than for TAS-102/Bevacizumab (30.4% vs. 2.9%, respectively; *p* < 0.001). *p*-values were calculated using Fisher’s exact test.

The impact of these toxicities on treatment continuity is visualized in Figure 4. A substantial divergence was observed in dose intensity management: 30.4% of patients in the Regorafenib group permanently discontinued treatment due to adverse events, compared to only 2.9% in the FTD/TPI plus bevacizumab group (*p* < 0.001). Furthermore, dose modifications were significantly more frequent in the Regorafenib arm (45.7%) than in the combination arm (11.4%, *p* < 0.001). These findings suggest that symptomatic toxicities in the Regorafenib group significantly compromised treatment persistence, whereas the asymptomatic nature of adverse events in the FTD/TPI plus bevacizumab group allowed sustained therapy.

### 3.4. Multivariate Analysis

To rigorously adjust for potential confounding variables and era effects, an expanded multivariate Cox proportional hazards analysis for TTF was performed (Table 3). Covariates were selected based on their established clinical prognostic significance in mCRC and potential impact on treatment selection. The model included treatment group, age, sex, ECOG performance status, liver metastasis, KRAS status, treatment line (>3rd vs. 3rd), year of treatment, prior anti-VEGF use, prior anti-EGFR use, peritoneal metastasis, number of metastatic sites, and baseline CEA (log-transformed).

After adjusting for these comprehensive baseline factors, the FTD/TPI plus bevacizumab regimen remained a significant independent predictor for favorable TTF, with a 56% reduction in the risk of treatment failure compared to regorafenib monotherapy (Hazard Ratio [HR] 0.44, 95% CI 0.22–0.91, *p* = 0.028).

Regarding other prognostic factors, liver metastasis was significantly associated with a higher risk of treatment failure (HR 2.06, 95% CI 1.12–3.79, *p* = 0.020), and higher baseline CEA levels (log-transformed) also predicted shorter TTF (HR 1.30, 95% CI 1.07–1.57, *p* = 0.007). Notably, prior anti-VEGF use was identified as an independent favorable prognostic factor (HR 0.44, 95% CI 0.20–0.98, *p* = 0.046). Other clinical factors, including age, sex, ECOG performance status, KRAS mutation status, and treatment line, were not significantly associated with treatment failure in this adjusted model.

### 3.5. Subgroup Analysis: Patients Aged ≥ 75 Years

To address the heterogeneity of the elderly population, a subgroup analysis was performed on patients aged ≥ 75 years (*n* = 18). Among these “oldest-old” patients, 10 received FTD/TPI plus bevacizumab and 8 received regorafenib. While the small sample size precluded robust statistical comparison, descriptive observations appeared consistent with the trends seen in the overall cohort. Patients in the FTD/TPI plus bevacizumab group demonstrated a manageable safety profile, with no unexpected severe adverse events or treatment-related mortality. This suggests that the combination strategy may represent a feasible therapeutic option even for patients in the advanced age bracket (≥75 years), provided that careful monitoring is maintained.

## 4. Discussion

In this retrospective study focusing on an elderly Asian population in a super-aged society, we compared the real-world effectiveness and safety of FTD/TPI plus bevacizumab versus regorafenib monotherapy. The results demonstrate that the FTD/TPI plus bevacizumab combination was associated with a significantly longer median Overall Survival (12.5 vs. 6.5 months, *p* = 0.039). Notably, while the univariate analysis for TTF showed a numerical trend, the expanded multivariate Cox regression identified the combination regimen as a significant independent predictor of favorable TTF (HR 0.44, *p* = 0.028), representing a 56% reduction in the risk of treatment failure after adjusting for key confounders, including treatment era and prior biologic use.

Regarding baseline performance status, ECOG PS was not identified as a statistically significant predictor of TTF in the multivariate analysis. This finding should be interpreted with caution, as it may reflect the limited sample size and potential measurement variability inherent in real-world assessments. However, from a clinical perspective, it is plausible that treatment outcomes in this heavily pretreated setting are driven not solely by baseline physical reserve, but also by the specific tolerability profile of the regimen. As observed in the regorafenib arm, unpredictable dose-limiting toxicities (e.g., HFSR) frequently precipitated discontinuation regardless of the patient’s initial performance status [6].

The observation that OS reached statistical significance while PFS did not is a common phenomenon in geriatric oncology, often attributed to the impact of post-progression survival [18]. This discrepancy suggests that the survival benefit of FTD/TPI plus bevacizumab represents a composite outcome, likely driven by a combination of direct antitumor efficacy, sustained tolerability, and the consequent preservation of access to subsequent therapies. Our data imply that by minimizing severe toxicity, this regimen helps maintain the functional status of elderly patients, adhering to the geriatric oncology principle of prioritizing tolerability to facilitate subsequent therapeutic interventions [3,19].

The stark contrast in treatment discontinuation rates observed in Figure 4 (30.4% in Regorafenib vs. 2.9% in FTD/TPI + Bev, *p* < 0.001) underscores a fundamental difference in treatment persistence between the two regimens [20]. While regorafenib-induced toxicities, particularly hand–foot skin reaction (58.7%) and gastrointestinal distress, are symptomatic and may impose a substantial burden on daily functioning, the toxicities associated with FTD/TPI plus bevacizumab are predominantly laboratory-based, such as neutropenia (48.6%) [7,21]. In clinical practice at a high-volume center, asymptomatic neutropenia was managed effectively through protocol-driven dose delays or G-CSF support, whereas symptomatic HFSR often led to permanent drug aversion among geriatric patients. This aligns with the ‘tolerability-first’ philosophy in geriatric oncology, where avoiding functional decline is as critical as achieving tumor control [22,23].

The optimal sequencing of late-line therapies remains a subject of intense debate. A recent real-world study from Taiwan (Chang et al., 2025) suggested that the sequence of “Regorafenib followed by FTD/TPI” might yield a superior median OS of 14.1 months compared to the reverse sequence [24]. While such findings support the strategy of utilizing regorafenib earlier while patients still possess adequate physiological reserve, our data highlights a critical limitation when applying this “general population logic” to a geriatric cohort.

While some real-world studies in the general population have suggested a survival benefit for the ‘regorafenib-first’ strategy, our findings indicate that this approach may be detrimental in the specific context of geriatric oncology [24,25]. The conventional ‘regorafenib-first’ rationale relies on patients retaining sufficient physiological reserve to tolerate subsequent therapies. However, in our elderly cohort, the ‘attrition rate’ during regorafenib treatment was alarmingly high. Only 17.4% of patients in the regorafenib arm were able to receive subsequent systemic therapy upon progression, compared to 45.7% in the FTD/TPI plus bevacizumab arm (*p* = 0.011). This suggests that regorafenib-induced symptomatic toxicities—specifically hand–foot skin reaction (58.7%)—often lead to a rapid decline in performance status, effectively closing the window for further treatment. In contrast, the ‘FTD/TPI + Bev-first’ strategy appears to act as a crucial bridge. By preserving patient tolerability, it allows a significantly higher proportion of elderly patients to access subsequent lines of therapy, avoiding the inherent risk of premature dropout associated with the upfront regorafenib strategy. This conclusion is further supported by the subgroup analysis of the REGOTAS study, which not only favored FTD/TPI in patients aged ≥ 65 years but also identified a significantly higher rate of discontinuation due to adverse events in the regorafenib arm [26].

Furthermore, our multivariate analysis confirmed that FTD/TPI plus bevacizumab is an independent predictor of superior TTF (HR 0.57, *p* = 0.025). This indicates that the combination regimen not only provides extended disease control but also possesses a favorable tolerability profile that supports a sustainable continuum of care [7,27].

A notable finding in our multivariate analysis was that prior anti-VEGF exposure served as an independent favorable prognostic factor for TTF (HR 0.44; *p* = 0.046). Although the FTD/TPI plus bevacizumab group had a higher proportion of patients with prior anti-VEGF use compared to the regorafenib group (Table 1), potential bias was rigorously addressed in our adjusted model. Crucially, even after controlling this variable, the survival benefit of the combination therapy remained statistically significant. This suggests that the therapeutic efficacy of FTD/TPI plus bevacizumab is intrinsic and independent of the patient’s prior history of biologic therapy.

The median OS of 12.5 months in the FTD/TPI plus bevacizumab cohort is notably favorable, aligning with recent large-scale real-world evidence from Japan [28]. This consistency suggests that Asian mCRC patients may derive substantial benefit from this combination, potentially due to ethnic differences in drug metabolism and tolerance compared to Western cohorts [24].

Several factors specific to the clinical setting further explain these superior outcomes. First, the high accessibility of healthcare in Taiwan, supported by the National Health Insurance (NHI) system, ensures that patients can be closely monitored and rapidly transitioned to subsequent treatment lines upon progression [29]. Second, the integration of Multidisciplinary Team (MDT) meetings at our institution allowed for prompt management of complications and effective utilization of palliative interventions, such as stenting or local radiotherapy, to preserve performance status [30]. Finally, the use of Shared Decision-Making (SDM) and proactive dose modification ensured that treatment was tailored to the individual’s physiological reserve, minimizing the risk of “death from toxicity” and allowing patients to achieve their maximal survival potential [31].

Regarding treatment selection, the choice between regimens was driven by a shared decision-making process weighing logistical convenience against toxicity profiles. Regorafenib was often favored by patients and caregivers seeking a purely oral regimen to minimize hospital commuting, provided they had adequate social support to manage dermatologic adverse events at home. Conversely, FTD/TPI plus bevacizumab was frequently prioritized for patients with frailty concerns or those explicitly wishing to avoid hand–foot skin reactions, accepting the trade-off of bi-weekly outpatient visits for bevacizumab infusions.

A recent multicenter study (Huang et al., JGO 2024) suggested that maintaining a daily dose of 120 mg for regorafenib is associated with improved survival in elderly mCRC patients, highlighting the importance of dose titration [32]. However, the current findings reveal a practical challenge: 30.4% of the elderly patients in this cohort discontinued regorafenib permanently due to intolerance (Figure 4), often before an optimal dose could be established.

In this context, FTD/TPI plus bevacizumab offers a distinct advantage. With a negligible discontinuation rate of 2.9% and a safety profile characterized mainly by asymptomatic neutropenia, this combination provides immediate tolerability without a complex “dose-finding” period. The findings of this study align with the landmark SUNLIGHT trial, which established FTD/TPI plus bevacizumab as a standard third-line therapy (OS: 10.8 vs. 7.5 months). Crucially, subgroup analyses of the SUNLIGHT trial confirmed that the survival benefit remains consistent in patients aged ≥ 65, without significant deterioration in Quality of Life (QoL) [7]. This is further supported by the Danish Phase 2 trial (Pfeiffer et al.), reinforcing the global applicability of this combination in maintaining a “Continuum of Care [27]”.

Despite the significant findings, several limitations should be acknowledged. First, the relatively small sample size from a single center might have limited the statistical power in univariate assessments; however, the robust significance achieved in the multivariate model for TTF underscores the strength of the treatment effect once baseline clinical variables were controlled. In this context, it is also important to recognize that TTF, as a composite endpoint, is inherently influenced by physician–patient interactions and shared decision-making thresholds regarding toxicity management. While this introduces an element of subjectivity compared to strict radiographic progression, we believe it accurately reflects the real-world complexities of maintaining elderly patients on active systemic therapy [10].

Second, a low rate of BRAF mutation testing and next-generation sequencing (NGS) analysis was observed in this cohort. This is primarily attributed to the high out-of-pocket costs associated with these advanced molecular diagnostics in Taiwan during the study period, as they were not fully covered by the national insurance system. Consequently, prognostic adjustment for BRAF status was not possible, and the potential impact of rare molecular subtypes on treatment response could not be exhaustively analyzed [33]. Therefore, our findings may not be fully generalizable to specific high-risk molecular subgroups.

Third, this study did not collect data regarding the socioeconomic status (SES) of the patients. In clinical practice, a patient’s economic capacity can significantly influence treatment choices, adherence to supportive care, and access to auxiliary treatments that are not fully reimbursed. Although the study drugs are covered by the National Health Insurance (NHI) system, potential disparities in SES between the treatment groups could lead to an unmeasured selection bias that may affect overall survival and quality of life outcomes [34].

Fourth, inherent limitations of the retrospective, non-randomized design must be acknowledged. Despite multivariate adjustment, the potential for confounding by indication cannot be fully excluded. Unmeasured factors, such as geriatric frailty (e.g., sarcopenia, G8 score), physician preference, imbalances in prior treatment history (e.g., bevacizumab exposure), or subtle differences in baseline performance status, might have influenced treatment selection. Furthermore, while all patients adhered to a standard follow-up protocol, minor variations in imaging intervals—potentially influenced by the frequency of hospital visits or admissions—could theoretically introduce interval censoring bias affecting PFS assessment.

Finally, the research was conducted within the context of Taiwan’s highly resource-intensive healthcare environment from 2019 to 2023. This study period spans evolving practice patterns, and potential treatment era effects cannot be completely ruled out. Under this framework, patients receive comprehensive and frequent medical monitoring, along with high accessibility to palliative care and multidisciplinary support [35,36]. While this exceptional level of care contributed to the favorable OS and PFS observed in this cohort, it may limit the generalizability of these findings to regions with different healthcare infrastructures and insurance models.

## 5. Conclusions

In conclusion, our study provides compelling real-world evidence that the combination of FTD/TPI plus bevacizumab is associated with longer overall survival and better treatment persistence in this cohort compared to regorafenib monotherapy for elderly Asian patients with refractory mCRC. The combination regimen was associated with significantly longer overall survival (12.5 months; *p* = 0.039) and exhibited a manageable safety profile characterized primarily by asymptomatic toxicities. Crucially, multivariate analysis identified the FTD/TPI plus bevacizumab regimen as a significant independent predictor of favorable time to treatment failure (*p* = 0.028). The observed tolerability profile was associated with significantly lower rates of premature treatment dropout—a common pitfall for the geriatric population—thereby potentially contributing to the maintenance of functional status and supporting a sustainable continuum of care that facilitates access to further lines of treatment. However, these findings require confirmation in larger, prospectively adjusted cohorts. In an increasingly aging society, these findings suggest that prioritizing a regimen with an optimal safety profile is paramount to maximizing survival outcomes while minimizing the symptomatic burden for vulnerable elderly patients.

## Figures and Tables

**Table 1:** Baseline Characteristics of the Elderly Study Population (Age ≥ 65).

Characteristic	Regorafenib (*n* = 46)	TAS-102 + Bev (*n* = 35)	*p*-Value
Age (years), Mean ± SD	70.2 ± 5.6	71.7 ± 6.2	0.208
Male Sex, *n* (%)	30 (65.2%)	20 (57.1%)	0.496
BMI (kg/m^2^), Mean ± SD	22.5 ± 3.3	21.9 ± 2.6	0.122
ECOG PS 0–1, *n* (%)	38 (82.6%)	29 (82.9%)	1.000
Comorbidities			
Hypertension (HTN)	26 (56.5%)	23 (65.7%)	0.493
Diabetes Mellitus (DM)	11 (23.9%)	14 (40.0%)	0.149
Hyperlipidemia	3 (6.5%)	4 (11.4%)	0.458
Primary Tumor Location (Left-sided)	36 (78.3%)	25 (71.4%)	0.604
Time from metastatic diagnosis, months Median (Range)	19.1 (0.0–77.5)	18.2 (0.0–74.4)	0.676
KRAS Mutant, *n* (%)	26 (56.5%)	16 (45.7%)	0.375
BRAF Status			
Mutant, *n* (%)	0 (0.0%)	0 (0.0%)	1.000
Wild-type, *n* (%)	2 (4.3%)	2 (5.7%)	
Unknown, *n* (%)	44 (95.7%)	33 (94.3%)	
Baseline CEA, Mean ± SD	158.2 ± 475.1	177.2 ± 613.8	0.170
Metastatic Sites			
Liver	27 (58.7%)	22 (62.9%)	0.819
Lung	18 (39.1%)	11 (31.4%)	0.494
Peritoneum	4 (8.7%)	8 (22.9%)	0.114
Prior History			
Primary Tumor Resection	40 (87.0%)	32 (91.4%)	0.725
Metastasectomy	26 (56.5%)	15 (42.9%)	0.266
Prior Anti-EGFR	10 (21.7%)	17 (48.6%)	0.017 *
Prior Bevacizumab	32 (69.6%)	34 (97.1%)	0.001 *
Any Subsequent Therapy	8 (17.4%)	16 (45.7%)	0.007 *
Systemic Therapy (Medicine)	7 (15.2%)	15 (42.9%)	0.011 *
Local Therapy (RT/Surgery)	1 (2.2%)	1 (2.9%)	1.000
Line of Therapy			0.680
3rd Line	19 (41.3%)	12 (34.3%)	
>3rd Line	27 (58.7%)	23 (65.7%)	

Abbreviations: SD, standard deviation; IQR, interquartile range; BMI, body mass index; ECOG PS, Eastern Cooperative Oncology Group performance status; HTN, hypertension; DM, diabetes mellitus; CEA, carcinoembryonic antigen; EGFR, epidermal growth factor receptor; WT, wild-type. Notes: Data are presented as mean ± SD or median (IQR) for continuous variables and number (percentage) for categorical variables. *p*-values were calculated using the Mann–Whitney U test for continuous variables (Age, BMI, CEA) and Pearson’s chi-square test or Fisher’s exact test for categorical variables. * Indicates a statistically significant difference (*p* < 0.05).

**Table 2:** Adverse Events (Safety Profile).

Adverse Event	Regorafenib (*n* = 46)		TAS-102 + Bev (*n* = 35)		*p*-Value (Any Grade)
	Any Grade	Grade ≥ 3	Any Grade	Grade ≥ 3	
Any Adverse Event	38 (82.6%)	12 (26.1%)	31 (88.6%)	13 (37.1%)	0.539
Hematologic					
Neutropenia	0 (0.0%)	0 (0.0%)	17 (48.6%)	11 (31.4%)	<0.001 *
Neutropenic fever	0 (0.0%)	0 (0.0%)	5 (14.3%)	5 (14.3%)	0.013 *
Anemia	1 (2.2%)	0 (0.0%)	2 (5.7%)	0 (0.0%)	0.575
Thrombocytopenia	3 (6.5%)	2 (4.3%)	2 (5.7%)	0 (0.0%)	1.000
Leukopenia	0 (0.0%)	0 (0.0%)	2 (5.7%)	0 (0.0%)	0.184
Non-hematologic					
Hand–foot skin reaction	27 (58.7%)	9 (19.6%)	0 (0.0%)	0 (0.0%)	<0.001 *
Fatigue	20 (43.5%)	4 (8.7%)	14 (40.0%)	2 (5.7%)	0.822
Diarrhea	9 (19.6%)	4 (8.7%)	0 (0.0%)	0 (0.0%)	0.009 *
Hypertension	2 (4.3%)	0 (0.0%)	3 (8.6%)	0 (0.0%)	0.647
Nausea/Vomiting	2 (4.3%)	1 (2.2%)	7 (20.0%)	0 (0.0%)	0.035 *
Liver dysfunction	8 (17.4%)	4 (8.7%)	0 (0.0%)	0 (0.0%)	0.009 *

Abbreviations: AE, adverse event; HFSR, hand–foot skin reaction; CTCAE, Common Terminology Criteria for Adverse Events. Notes: Data are presented as number (percentage) of patients. Adverse events were graded according to NCI-CTCAE version 5.0. *p*-values were calculated using Fisher’s exact test comparing the incidence of any-grade adverse events between the two treatment groups. * indicates a statistically significant difference (*p* < 0.05).

**Table 3:** Cox Proportional Hazards Model for TTF.

Variable	Univariate HR (95% CI)	*p*-Value	Multivariate HR (95% CI)	*p*-Value
Treatment Group (FTD/TPI + Bev vs. Regorafenib)	0.77 (0.49–1.20)	0.241	0.44 (0.22–0.91)	0.028 *
Age (≥75 vs. <75 years)	1.13 (0.67–1.91)	0.655	1.45 (0.80–2.62)	0.224
Sex (Male vs. Female)	1.03 (0.65–1.61)	0.906	1.03 (0.60–1.77)	0.912
ECOG PS (≥2 vs. 0–1)	0.68 (0.37–1.27)	0.232	0.78 (0.35–1.71)	0.527
Liver Metastasis (Yes vs. No)	1.52 (0.96–2.39)	0.073	2.06 (1.12–3.79)	0.020 *
KRAS Status (Mutant vs. Wild-type)	1.04 (0.67–1.61)	0.866	1.22 (0.55–2.68)	0.625
Treatment Line (>3rd- vs. 3rd-line)	1.08 (0.68–1.70)	0.749	1.34 (0.77–2.32)	0.296
Year of Treatment (Continuous)	1.02 (0.89–1.17)	0.781	1.24 (1.00–1.54)	0.052
Prior anti-VEGF (Yes vs. No)	0.56 (0.32–0.99)	0.046 *	0.44 (0.20–0.98)	0.046 *
Prior anti-EGFR (Yes vs. No)	0.93 (0.58–1.48)	0.761	0.87 (0.37–2.01)	0.739
Peritoneal Metastasis (Yes vs. No)	1.20 (0.64–2.23)	0.570	1.63 (0.70–3.81)	0.260
Baseline CEA (Log-transformed)	1.10 (0.95–1.29)	0.205	1.30 (1.07–1.57)	0.007 *
No. of Metastatic Sites (≥2 vs. 1)	0.99 (0.60–1.64)	0.975	0.89 (0.50–1.57)	0.682

Abbreviations: HR, hazard ratio; CI, confidence interval; ECOG PS, Eastern Cooperative Oncology Group performance status; CEA, carcinoembryonic antigen; VEGF, vascular endothelial growth factor; EGFR, epidermal growth factor receptor. * *p* < 0.05.

## Data Availability

The data presented in this study are available on request from the corresponding author. The data are not publicly available due to patient privacy regulations and ethical restrictions.

## Abbreviations

The following abbreviations are used in this manuscript:

mCRC	Metastatic Colorectal Cancer
FTD/TPI	Trifluridine/Tipiracil
OS	Overall Survival
PFS	Progression-Free Survival
TTF	Time to Treatment Failure
HFSR	Hand–Foot Skin Reaction
ECOG	Eastern Cooperative Oncology Group
HR	Hazard Ratio
CI	Confidence Interval
AE	Adverse Event
TCVGH	Taichung Veterans General Hospital

## References

[1] Bray F., Laversanne M., Sung H., Ferlay J., Siegel R.L., Soerjomataram I., Jemal A. (2024). Global cancer statistics 2022: GLOBOCAN estimates of incidence and mortality worldwide for 36 cancers in 185 countries. CA Cancer J. Clin..

[2] Huemer F., Dunkl C., Rinnerthaler G., Schlick K., Heregger R., Emmanuel K., Neureiter D., Klieser E., Deutschmann M., Roeder F. (2023). Management of metastatic colorectal cancer in patients ≥ 70 years—A single center experience. Front. Oncol..

[3] Wang X., Ma R., Hou T., Xu H., Zhang C., Ye C. (2025). Robotic versus laparoscopic surgery for colorectal cancer in older patients: A systematic review and meta-analysis. Minim. Invasive Ther. Allied Technol..

[4] Grothey A., Van Cutsem E., Sobrero A., Siena S., Falcone A., Ychou M., Humblet Y., Bouché O., Mineur L., Barone C. (2013). Regorafenib monotherapy for previously treated metastatic colorectal cancer (CORRECT): An international, multicentre, randomised, placebo-controlled, phase 3 trial. Lancet.

[5] Li J., Qin S., Xu R., Yau T.C., Ma B., Pan H., Xu J., Bai Y., Chi Y., Wang L. (2015). Regorafenib plus best supportive care versus placebo plus best supportive care in Asian patients with previously treated metastatic colorectal cancer (CONCUR): A randomised, double-blind, placebo-controlled, phase 3 trial. Lancet Oncol..

[6] Ducreux M., Petersen L.N., Öhler L., Bergamo F., Metges J.P., de Groot J.W., Wang J.Y., García Paredes B., Dochy E., Fiala-Buskies S. (2019). Safety and effectiveness of regorafenib in patients with metastatic colorectal cancer in routine clinical practice in the prospective, observational CORRELATE study. Eur. J. Cancer.

[7] Prager G.W., Taieb J., Fakih M., Ciardiello F., Van Cutsem E., Elez E., Cruz F.M., Wyrwicz L., Stroyakovskiy D., Pápai Z. (2023). Trifluridine-Tipiracil and Bevacizumab in Refractory Metastatic Colorectal Cancer. N. Engl. J. Med..

[8] Ness R.M., Llor X., Abbass M.A., Bishu S., Chen C.T., Cooper G., Early D.S., Friedman M., Fudman D., Giardiello F.M. (2024). NCCN Guidelines^®^ Insights: Colorectal Cancer Screening, Version 1.2024. JNCCN J. Natl. Compr. Cancer Netw..

[9] World Medical Association (2013). Declaration of Helsinki: Ethical principles for medical research involving human subjects. JAMA.

[10] Boland P.A., McEntee P.D., Murphy E., Singaravelu A., Tuynmann J.B., Arezzo A., Aigner F., Burke J.P., Cahill R.A. (2025). Local excision of rectal neoplasia: A real-world survey of current practices and perspectives. Minim. Invasive Ther. Allied Technol..

[11] Bekaii-Saab T.S., Ou F.S., Ahn D.H., Boland P.M., Ciombor K.K., Heying E.N., Dockter T.J., Jacobs N.L., Pasche B.C., Cleary J.M. (2019). Regorafenib dose-optimisation in patients with refractory metastatic colorectal cancer (ReDOS): A randomised, multicentre, open-label, phase 2 study. Lancet Oncol..

[12] Eisenhauer E.A., Therasse P., Bogaerts J., Schwartz L.H., Sargent D., Ford R., Dancey J., Arbuck S., Gwyther S., Mooney M. (2009). New response evaluation criteria in solid tumours: Revised RECIST guideline (version 1.1). Eur. J. Cancer.

[13] Pillay B., Wootten A.C., Crowe H., Corcoran N., Tran B., Bowden P., Crowe J., Costello A.J. (2016). The impact of multidisciplinary team meetings on patient assessment, management and outcomes in oncology settings: A systematic review of the literature. Cancer Treat. Rev..

[14] Freites-Martinez A., Santana N., Arias-Santiago S., Viera A. (2021). Using the Common Terminology Criteria for Adverse Events (CTCAE—Version 5.0) to Evaluate the Severity of Adverse Events of Anticancer Therapies. Actas Dermosifiliogr.

[15] Mayer R.J., Van Cutsem E., Falcone A., Yoshino T., Garcia-Carbonero R., Mizunuma N., Yamazaki K., Shimada Y., Tabernero J., Komatsu Y. (2015). Randomized trial of TAS-102 for refractory metastatic colorectal cancer. N. Engl. J. Med..

[16] Kaplan E.L., Meier P. (1958). Nonparametric Estimation from Incomplete Observations. J. Am. Stat. Assoc..

[17] Cox D.R. (1972). Regression Models and Life-Tables. J. R. Stat. Society. Ser. B Methodol..

[18] Petrelli F., Barni S. (2013). Correlation of progression-free and post-progression survival with overall survival in advanced colorectal cancer. Ann. Oncol..

[19] Papamichael D., Audisio R.A., Glimelius B., de Gramont A., Glynne-Jones R., Haller D., Köhne C.H., Rostoft S., Lemmens V., Mitry E. (2015). Treatment of colorectal cancer in older patients: International Society of Geriatric Oncology (SIOG) consensus recommendations 2013. Ann. Oncol..

[20] Sonbol M.B., Benkhadra R., Wang Z., Firwana B., Walden D.J., Mody K., Hubbard J.M., Murad M.H., Ahn D.H., Bekaii-Saab T. (2019). A Systematic Review and Network Meta-Analysis of Regorafenib and TAS-102 in Refractory Metastatic Colorectal Cancer. Oncologist.

[21] Krishnamoorthy S.K., Relias V., Sebastian S., Jayaraman V., Saif M.W. (2015). Management of regorafenib-related toxicities: A review. Ther. Adv. Gastroenterol..

[22] Fakih M., Ciardiello F., Prager G.W., Élez E., Calleja E., Caussé-Amellal N., Taieb J., Van Cutsem E. (2025). Managing adverse events in patients with metastatic colorectal cancer receiving trifluridine/tipiracil in combination with bevacizumab. ESMO Gastrointest. Oncol..

[23] Purcell I., Potter V., Bell L., Brooks H., Gangadhara S., Kunene V., Teo P.J., Iqbal Malik M. (2025). Granulocyte Colony Stimulating Factor in the Prevention of Chemotherapy-Induced Neutropenia in Patients With Colorectal Cancer Receiving Trifluridine/Tipiracil: A Real-World UK Retrospective Review. Cancer Control.

[24] Chang Y.W., Kuo C.N., Chang C.L., Hsu J.C., Ko Y. (2025). Sequential Treatment of Metastatic Colorectal Cancer in Taiwan: Real-World Evidence From Regorafenib and Trifluridine/Tipiracil Use. J. Gastroenterol. Hepatol..

[25] Signorelli C., Calegari M.A., Basso M., Anghelone A., Lucchetti J., Minelli A., Angotti L., Zurlo I.V., Schirripa M., Chilelli M.G. (2023). Treatment Settings and Outcomes with Regorafenib and Trifluridine/Tipiracil at Third-Line Treatment and beyond in Metastatic Colorectal Cancer: A Real-World Multicenter Retrospective Study. Curr. Oncol..

[26] Moriwaki T., Fukuoka S., Taniguchi H., Takashima A., Kumekawa Y., Kajiwara T., Yamazaki K., Esaki T., Makiyama C., Denda T. (2018). Propensity Score Analysis of Regorafenib Versus Trifluridine/Tipiracil in Patients with Metastatic Colorectal Cancer Refractory to Standard Chemotherapy (REGOTAS): A Japanese Society for Cancer of the Colon and Rectum Multicenter Observational Study. Oncologist.

[27] Pfeiffer P., Yilmaz M., Möller S., Zitnjak D., Krogh M., Petersen L.N., Poulsen L., Winther S.B., Thomsen K.G., Qvortrup C. (2020). TAS-102 with or without bevacizumab in patients with chemorefractory metastatic colorectal cancer: An investigator-initiated, open-label, randomised, phase 2 trial. Lancet Oncol..

[28] Kagawa Y., Shinozaki E., Okude R., Tone T., Kunitomi Y., Nakashima M. (2023). Real-world evidence of trifluridine/tipiracil plus bevacizumab in metastatic colorectal cancer using an administrative claims database in Japan. ESMO Open.

[29] Wu T.Y., Majeed A., Kuo K.N. (2010). An overview of the healthcare system in Taiwan. Lond. J. Prim. Care.

[30] Hsu Y.H., Kung P.T., Wang S.T., Fang C.Y., Tsai W.C. (2016). Improved patient survivals with colorectal cancer under multidisciplinary team care: A nationwide cohort study of 25,766 patients in Taiwan. Health Policy.

[31] Rostoft S., van den Bos F., Pedersen R., Hamaker M.E. (2021). Shared decision-making in older patients with cancer—What does the patient want?. J. Geriatr. Oncol..

[32] Huang J., Gong C., Jiang Z., Qu W., Sun Y., Teo N.Z., Zhang W., Yang L., Zhao Y., Zhou A. (2024). Regorafenib monotherapy as the later-line treatment for elderly patients with metastatic colorectal cancer: A multicenter real-world study. J. Gastrointest. Oncol..

[33] Huang C.-Y., Huang W.-K., Yeh K.-Y., Chang J.W.-C., Lin Y.-C., Chou W.-C. (2025). Integrating comprehensive genomic profiling in the management of oncology patients: Applications and challenges in Taiwan. Biomed. J..

[34] Kuo W.Y., Hsu H.S., Kung P.T., Tsai W.C. (2021). Impact of Socioeconomic Status on Cancer Incidence Risk, Cancer Staging, and Survival of Patients with Colorectal Cancer under Universal Health Insurance Coverage in Taiwan. Int. J. Environ. Res. Public Health.

[35] Lee Y.C., Huang Y.T., Tsai Y.W., Huang S.M., Kuo K.N., McKee M., Nolte E. (2010). The impact of universal National Health Insurance on population health: The experience of Taiwan. BMC Health Serv. Res..

[36] Po-Chang L., Lee P.-C., Wang J.T.-H., Chen T.-Y., Peng C.-H. (2022). Introduction to the National Health Insurance of Taiwan. Digital Health Care in Taiwan: Innovations of National Health Insurance.

